# Economic Implications of Audiology Student Debt for Workforce Sustainability

**DOI:** 10.1055/s-0046-1825789

**Published:** 2026-10-06

**Authors:** Amyn M. Amlani, Jaime S. Schulang

**Affiliations:** 1 Department of Communication Sciences and Disorders, College of Allied Health, University of Oklahoma Health Sciences Center, Oklahoma City, Oklahoma, United States; 2 Office of Financial Aid, Enrollment Management, Drexel University, Drexel University Elkins Park Campus, Elkins Park, Pennsylvania, United States

**Keywords:** student debt, debt-to-income ratio, workforce sustainability, Doctor of Audiology (AuD)

## Abstract

Rising educational costs and comparatively modest early-career wages have created a structural return-on-investment problem for the Doctor of Audiology (AuD) degree, with implications extending beyond individual finances to workforce sustainability and access to care. This article examines the economic burden of audiology training within the broader context of U.S. graduate student debt, using debt-to-income (DTI) analysis to compare early-career AuD professionals with speech-language pathology peers under otherwise identical financial assumptions. Findings indicate that AuD graduates carry substantially higher back-end DTI ratios and retain markedly less discretionary income, driven by higher average graduate debt, exclusion from favorable federal lending programs available to comparable health professions, and an uncompensated fourth-year clinical externship that concentrates the full financial burden of that year into a period with no or insufficient offsetting income. Recent federal policy changes under the One Big Beautiful Bill Act (OBBBA)–including new aggregate borrowing caps and an unsettled definition of "professional degree" programs–introduce additional uncertainty for future cohorts. The article outlines institutional, federal, and state-level reforms, with particular attention to externship compensation models, as levers to reduce debt accumulation while strengthening workforce entry, retention, and geographic distribution. Meaningful improvement requires alignment between training cost and professional earning potential, not cost containment alone.

## Introduction

In the United States, earning a graduate degree has long been regarded as a sound personal and professional investment. For many pursuing the Doctor of Audiology (AuD), however, that assumption is increasingly strained by a convergence of structural economic pressures. Prior articles in this issue have established two foundational conditions: audiologists' wages have not risen commensurately with the transition to doctoral-level training (Economics of Audiology Wages), and audiology's professional classification—across scope of practice, reimbursement, and federal program eligibility (Audiology's Nomenclature and Taxonomy)—leaves the profession structurally disadvantaged relative to peer health disciplines. This article examines what happens when high training costs meet those suppressed and constrained earning conditions. The result is a return on investment (ROI) problem that is not incidental or correctable by individual financial planning, but structural—one that shapes who enters the profession, where they practice, and how long they remain.

Educational debt associated with audiology training extends beyond individual financial obligations. Accrued debt is associated with decisions regarding employment setting, geographic location, career trajectory, and the timing of major life milestones and may influence professional longevity. For a profession already confronting workforce constraints, geographic maldistribution, and expanding demand for hearing and balance services, these economic pressures carry implications for access to care, as well as long-term workforce stability of the profession.

Within a healthcare environment increasingly focused on value, accountability, and efficient workforce deployment, audiology must critically examine whether its current educational and training model produces returns—both personal and professional—that are proportionate to its cost. Evaluating the ROI of audiology education is, therefore, essential for prospective students, academic programs, accrediting bodies, and professional leaders seeking to ensure a sustainable, equitable, and future-ready audiology workforce. This article serves as a bridge within the issue's larger argument: it receives the empirical findings on wage suppression and professional misclassification established in earlier articles and translates their consequences into the lived financial reality of AuD graduates, before handing off to the health economics, education modernization, and workforce roadmap articles that follow. While the analyses presented here also carry implications for broader policy discussions, the primary aim is to inform decision-making within the profession itself.

### Part I. The Economic Context of Student Debt

Student loan debt has become one of the most consequential forms of consumer borrowing in the United States. Educational borrowing is incurred early in adulthood, is often nondischargeable, and is undertaken with the expectation of long-term economic return. Today, approximately half of individuals completing postsecondary education graduate with student loan debt, making it a routine feature of workforce entry rather than an exceptional circumstance. 1

At the national level, student debt now represents the second-largest category of household consumer debt—exceeded only by mortgages and surpassing both credit card and auto loan balances. 2 This growth reflects not only increased participation in higher education but also a structural shift in how education is financed. Over time, public subsidies have declined relative to total educational expenditures, transferring a growing share of cost and risk from institutions and governments to individual borrowers. 3 As a result, student debt has become a durable financial obligation that shapes economic behavior well beyond graduation.

The implications of this shift extend far beyond loan repayment. High debt burdens influence household formation, geographic mobility, savings and retirement behavior, and career decision-making—particularly during the first decade of professional life. These effects are most pronounced in fields where early-career wages are modest or wage growth is constrained, as prolonged repayment obligations compress disposable income and limit financial flexibility. 1

When educational costs rise faster than expected earnings, the ROI of a degree becomes increasingly uncertain. This misalignment has implications not only for individuals but also for professions that depend on a stable and replenishing workforce. Debt burden can shape who enters a profession, where they are able to practice, and how long they remain in the field, making student debt a structural workforce issue rather than a purely personal financial challenge.

Within this broader economic context, the AuD provides a particularly salient case study. As a 4-year clinical doctorate with substantial tuition requirements and limited access to subsidized training mechanisms, audiology sits at the intersection of rising educational costs and comparatively modest early-career compensation. The sections that follow examine how national trends in higher education finance intersect with the specific structure of audiology training, shaping both individual economic outcomes and the profession's long-term workforce capacity.

#### Historical Trends in Higher Education Costs

According to the National Center for Education Statistics (NCES), average “nominal” undergraduate tuition and required fees at U.S. public and private institutions increased by more than 160% between the 2000 to 2001 and 2021 to 2022 academic years, rising from $5,377 to $14,306. 4 Over the same period, cumulative consumer inflation—as measured by the Consumer Price Index for All Urban Consumers (CPI-U)—increased by approximately 57%. 5 As seen in Fig. 1 , this divergence indicates that growth in undergraduate costs has substantially outpaced general inflation, placing disproportionate financial pressure on students and borrowers.

**Fig. 1:**
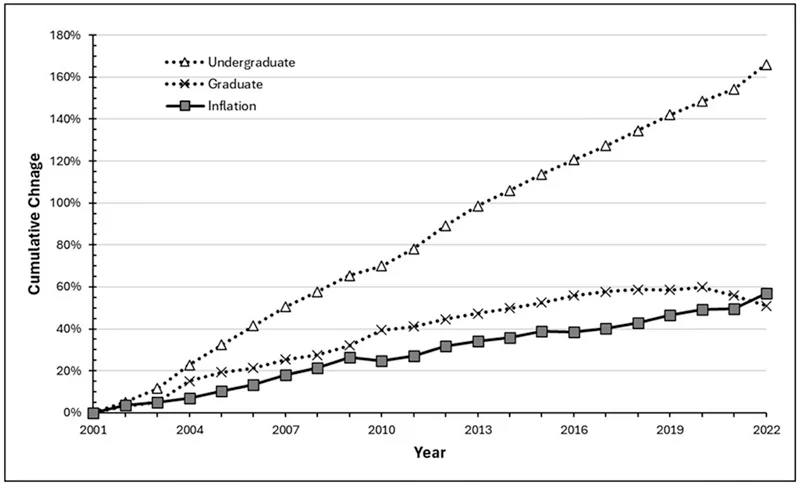
Percentage change in average nominal undergraduate and graduate tuition at U.S. institutions relative to cumulative consumer inflation (CPI-U), 2001 to 2022. Data from NCES 4 6 and Bureau of Labor Statistics (BLS). 5

Conversely, NCES data indicate that average “nominal” graduate tuition and required fees increased by approximately 51% between the 2000 and 2001 academic year ($13,578) and the 2021 to 2022 academic year ($20,513), 6 as shown in Fig. 1 . Although the proportional increase in graduate tuition is lower than that observed at the undergraduate level and relative to cumulative consumer inflation, the absolute cost burden—particularly for doctorates in health-related fields—remains substantially higher. These trends reflect a broader structural shift in how higher education is financed, characterized by small, incremental growth in population wages and declining per-student public subsidies; together, there is an increasing reliance on individual borrowing to offset institutional costs. 5 For clinical doctorates such as audiology, this shift places a disproportionate burden on students, as program length and clinical training requirements constrain opportunities for subsidized support.

Several interrelated economic and policy developments have contributed to this upward cost trajectory. 7 First, state appropriations for public colleges and universities have declined on a real, per-student basis over the past two decades, requiring institutions to rely more heavily on tuition revenue to sustain operations. Second, the expansion of federal student loan programs—particularly the availability of unsubsidized loans—has increased students' borrowing capacity, reducing short-term price sensitivity and enabling tuition increases with limited immediate market resistance. Third, growing investments in campus infrastructure, administrative staffing, information technology, accreditation, and regulatory compliance have elevated institutional operating costs, many of which are ultimately passed on to students through tuition and fees.

These historical cost trends are especially consequential for health-profession education, which typically requires extensive laboratory instruction, simulation resources, and supervised clinical training. Clinical doctorates—including the AuD—often involve small cohort sizes resulting in low student-to-faculty ratios and reliance on external clinical placements, all of which increase program delivery costs. As with many clinically intensive graduate and professional programs, the full-time structure of audiology education limits access to teaching and research assistantships common in nonclinical degrees. However, unlike several other clinical doctorates that incorporate paid clinical training, higher federal loan caps, or structured repayment incentives, audiology education remains predominantly tuition-dependent, leaving most students responsible for financing the full cost of training through loans.

For students pursuing the AuD degree, these broader economic patterns translate into substantial personal monetary responsibility. As tuition continues to rise while institutional support remains constrained, the gap between educational cost and affordable financing widens, compelling increased reliance on federal and private student loans. Understanding these historical cost trends is, therefore, essential for evaluating the ROI of audiology education and for assessing the long-term sustainability of the profession's workforce pipeline.

#### The Audiology Context: Costs, Structure, and the 4-Year Doctorate

At its inception, the AuD was established to elevate clinical training, expand diagnostic competence, and align audiology with other health professions that require a professional doctorate for entry to practice. At the same time, it introduced a structural reality with significant financial implications: a 4-year, full-time educational model with comparatively limited opportunities for subsidized training relative to many other clinical doctorates.

At most institutions, AuD training consists of 3 years of didactic coursework, laboratory instruction, and supervised clinical rotations, followed by a fourth-year full-time externship. Relative to peer health professions—such as physician assistant (PA) education and select physical therapy (PT) training models—AuD students receive comparatively limited direct financial support during graduate training. Whereas many PA programs and some PT pathways incorporate tuition remission, stipends, paid clinical training, or structured loan repayment incentives, 8 9 audiology education remains largely tuition-dependent, with limited availability of assistantships, stipends, or externally funded clinical placements. Importantly, this distinction reflects differences in program cost structure rather than program scale: AuD programs typically operate with smaller cohort sizes, which increase per-student instructional cost and limit the ability to offset expenses through enrollment volume. In the absence of institutional subsidies or external funding mechanisms, these higher per-student costs are passed directly to trainees, contributing to higher cumulative debt among AuD graduates.

Three interrelated features of the current AuD model are particularly salient. First, clinical training occurs largely through off-campus placements in which students function as supervised learners rather than salaried trainees, yet remain responsible for tuition, university fees, and living expenses throughout the program. The fourth-year externship represents the most visible–and often most contested–expression of this structure. During this year, students commonly pay full–time tuition–often equivalent to 9 to 12 academic credits per term–while receiving the majority of their clinical supervision from externally licensed audiologists who typically receive no direct compensation from the university.

Students themselves, however, are not uniformly unpaid during this year. A historical accommodation tracing back to audiology's transition from the post-graduate Clinical Fellowship Year (CFY) to the current in-program externship model allows some sites to offer "stipends" to offset the extern's cost of living, even though the student remains under supervision rather than independently licensed – a practice with no clear parallel in comparably structured clinical externships in other health professions. For example, a 10–year analysis of 262 AuD externship placements (2013–2022), 10 found that roughly 73% of externs received some compensation, with mean annual stipends ranging from approximately $14,000 to $21,000 depending on site type. Compensation is far from uniform, however: hospital sites, the most frequently selected placement type, paid the least and were the most likely to pay nothing at all (43% uncompensated), while private practice and medical office placements paid more consistently and at higher levels.

It is important to acknowledge that, in programs where fourth year tuition is reduced, costs are frequently redistributed to earlier years such that total degree tuition remains unchanged. Thus, the externship year does not necessarily increase “total” program cost, but rather concentrates the financial burden during a period when students have limited institutional contact, minimal access to financial support, and, for the roughly one in four externs placed at non-paying sites, no opportunity for earned income. Because tuition and living expenses persist throughout this fulltime clinical year–regardless of how costs are allocated across the curriculum–AuD students often rely disproportionately on Direct Graduate PLUS Loans, amplifying interest accrual and long–term repayment burden.

From a workforce and ROI perspective, the issue is, therefore, not simply the distribution of tuition across years, but the alignment between educational cost, instructional delivery, and financial accountability during the externship period. When substantial tuition is charged during a year in which instruction is delivered primarily by uncompensated external clinicians, and student compensation – where it exists at all – is informal, site–dependent, and unstandardized rather than tied to any wage floor or clinical productivity standard, the economic value proposition of the clinical doctorate warrants closer scrutiny in terms of transparency, efficiency, and sustainability.

Second, the opportunity cost of the AuD pathway further constrains economic viability. Unlike medical residents or certain allied health trainees who earn regulated, standardized salaries during advanced clinical training, audiology students receive – at best – an unstandardized stipend that, even at its higher site-type averages, falls well short of a living wage and is unavailable to roughly a quarter of externs outright. Over 4 years of training, many students forgo full-time income, delay retirement contributions and asset accumulation, and accrue interest on borrowed funds. These cumulative direct and indirect costs materially reduce the economic return of the degree, particularly during the early-career period when wages are modest and loan repayment obligations are highest.

Third, substantial variability in program costs across institutions compounds these pressures. Tuition differs markedly by public versus private status, in-state versus out-of-state enrollment, and geographic region. Some students enter professional programs with total tuition below approximately $80,000, while others face costs approaching or exceeding $150,000, exclusive of undergraduate debt. The decentralized nature of audiology education means that students often make enrollment decisions without standardized or transparent data regarding total debt burden, externship compensation, graduation outcomes, or long-term earning trajectories.

Taken together, these structural features explain why the financial burden of audiology education is borne primarily by students and persists well beyond graduation. The interaction between program length, high per-student instructional cost, limited institutional support, and an externship year in which compensation is inconsistent, site-dependent, and structurally unstandardized shifts both direct and opportunity costs onto trainees, shaping their financial position at workforce entry. As a result, the economic impact of audiology education is most clearly observed in early-career debt-to-income (DTI) ratios and constrained financial flexibility—issues examined in the following section.

#### Economic Burden and Debt-to-Income Ratios in Audiology

Understanding the economic burden of audiology education requires more than an accounting of total student loan balances; it also requires assessing how those obligations affect day-to-day financial stability and long-term economic mobility. The DTI ratio is one of the most widely used indicators of financial capacity, credit risk, and affordability. 11 It is a core metric employed by lenders when evaluating eligibility for mortgages, auto loans, and other forms of consumer credit. Within the context of audiology, DTI provides a pragmatic framework for evaluating the financial strain experienced by early-career AuD professionals relative to peers in comparable health professions.

##### Debt-to-Income Ratio Framework

DTI ratios compare an individual's total monthly debt obligations to gross monthly income. A higher DTI reflects a greater proportion of income committed to fixed debt payments, reducing financial flexibility and increasing perceived lending risk. Lenders typically evaluate two forms of DTI:

- “Front-end ratio (housing ratio)”: monthly housing costs—including rent or mortgage payments, interest, property taxes, insurance, and private mortgage insurance—relative to gross monthly income. Conventional underwriting guidelines generally prefer ratios below 28%, although thresholds up to approximately 31 to 36% may be permitted depending on borrower profile.
- “Back-end ratio”: the sum of monthly housing costs and all other recurring debt obligations (e.g., student loans, auto loans, credit card minimum payments, alimony) relative to gross monthly income. Preferred thresholds are typically below 36%, with upper limits commonly ranging from 43 to 50%, above which borrowers are considered higher risk.

Using these financial benchmarks, early-career professionals with higher levels of educational debt face tighter financial constraints that can influence borrowing capacity, housing access, and long-term economic mobility.

##### DTI Example: Early-Career AuD versus SLP Professionals

Table 1 presents a hypothetical comparison of DTI ratios for early-career AuD and speech-language pathology (SLP) professionals. The example assumes identical entry-level annual salaries ($78,000), housing expenses, consumer debt obligations, and credit histories. The distinguishing variable is average graduate-level student loan debt: approximately $96,175 for AuD graduates and $43,644 for SLP graduates. 1

**Table 1:** Hypothetical comparison of front-end and back-end DTI between early-career AUD and SLP professionals 4

Front-end DTI		
	AUD	SLP
Gross monthly income (annual salary of $78,000)	$6,500	$6,500
Monthly housing rent	$1,463	$1,463
Monthly renter's insurance	$20	$20
Total debt	$1,483	$1,483
DTI—front end (monthly housing expenses/monthly gross income)	23%	23%
Back-end DTI		
	AUD	SLP
Gross monthly income (annual salary of $78,000)	$6,500	$6,500
Monthly housing rent	$1,463	$1,463
Monthly renter's insurance	$20	$20
Monthly minimum credit card payment	$212	$212
Monthly minimum auto payment	$512	$512
Monthly student loan payment	$717	$325
Total debt	$2,924	$2,532
DTI—back end [(monthly housing expenses + debts)/monthly gross income]	45%	39%

Note: See text for assumptions and details **.**

Applying identical loan terms (20-year repayment at 6.5% interest), the AuD professional's monthly student loan payment exceeds twice that of the SLP professional ($717 vs. $325). This difference materially alters back-end DTI calculations despite equivalent income and noneducational debt.

Although both professionals fall within preferred limits for front-end DTI, their back-end ratios diverge meaningfully. The SLP professional remains within commonly accepted underwriting thresholds, whereas the AuD professional exceeds the typical upper bound, placing them in a higher-risk lending category. In practice, such profiles are more likely to encounter higher interest rates, reduced borrowing limits, or additional lending conditions. Over time, these constraints can compound, limiting access to home ownership, delaying asset accumulation, and constraining long-term financial stability.

##### DTI Across Health Professions

To contextualize audiology within the broader landscape of health professions, Fig. 2 depicts estimated graduate-only student loan debt across six professions—optometry (OPT), PA, occupational therapy (OT), PT, SLP, and audiology (AuD)—for graduating cohorts between 2010 and 2022. 12 Undergraduate borrowing is excluded to isolate debt attributable solely to graduate or professional training.

**Fig. 2:**
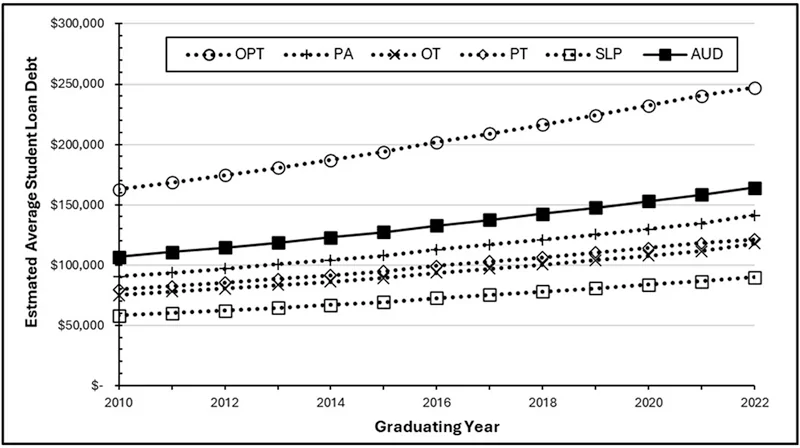
Estimated average student loan debt by graduating year across selected health professions, accumulated during graduate or professional education only (undergraduate debt excluded). See text for data sources and assumptions.

To quantify longitudinal trends, linear regression models were fitted for each profession, with graduating year specified as the independent variable ( *x* ) and mean graduate-level debt as the dependent variable ( *y* ). The slope of each regression line ( *m* ) represents the estimated annual increase in graduate-only student debt (in U.S. dollars per year).

As shown in Table 2 , substantial differences in debt accumulation trajectories are observed across professions. OPT demonstrates the highest annual growth in graduate-only student debt ($5,886 per year), followed by AuD ($3,515 per year), while SLP exhibits the lowest annual increase ($1,392 per year). For all professions, the regression variable showed a good fit, where 99% of the variance in student debt (dependent variable) was explained by profession (independent variable).

**Table 2:** Linear regression estimates of annual change in graduate- or professional-school student loan debt across selected health professions

Profession	Linear regression ( *y = mx* + *b* )	Model fit ( *R* ^2^ )
OPT	y = 5,886 *x* + 129,694	0.99
PA	y = 2,810 *x* + 61,455	0.99
OT	y = 2,090 *x* + 47,158	0.99
PT	y =2,309 *x* + 51,388	0.99
SLP	y = 1,392 *x* + 30,978	0.99
AuD	y = 3,515 *x* + 76,835	0.99

Note: Slopes ( *m* ) represent estimated dollar increase per graduating year **.**

##### Affordability and Disposable Income

DTI ratios directly influence affordability—that is, the proportion of income remaining after meeting fixed financial obligations. Although DTI is traditionally used by lenders to assess credit risk, it also provides a useful framework for approximating functional disposable income available for day-to-day expenses, savings, and long-term financial stability.

Back-end DTI, as applied in lending decisions, reflects the proportion of “gross income” allocated to contractual debt obligations (e.g., housing, student loans, auto loans, credit cards) and does not incorporate taxes, retirement savings, or other nondiscretionary living expenses. To extend the analytic usefulness of DTI for workforce and quality-of-life considerations, Table 3 decomposes gross income by combining back-end DTI with estimated income taxes and baseline retirement contributions. This approach provides an approximation—rather than a precise accounting—of disposable income available to early-career professionals after meeting core financial obligations.

**Table 3:** Decomposition of gross income into debt obligations and nondiscretionary expenses for early-career AuD and SLP professionals

AuD		SLP
Back-end DTI (debt obligations only)	45%	39%
Income taxes (includes state/local taxes)	30%	30%
Retirement savings	6%	6%
Total obligations	81%	75%
Remaining disposable income	19%	25%

Note: Percentages are illustrative and based on assumptions described in the text.

Under these assumptions, the AuD professional retains approximately 19% of gross income as discretionary income, compared with 25% for the SLP professional. This difference has meaningful implications for financial flexibility, savings behavior, and tolerance for unexpected expenses.

If both professionals seek to purchase a home under comparable financing assumptions, housing-related costs further reduce disposable income by an estimated 12%. This estimate reflects the replacement of current rental expenses ($1,483 per month; Table 1 ) with an approximate $780 monthly increase associated with mortgage principal and interest, homeowner's insurance, and private mortgage insurance. Under these conditions, postpurchase disposable income declines to approximately 7% for the AuD professional (19–12%) and 13% for the SLP professional (25–12%).

Even under idealized conditions—identical salaries, similar credit profiles, and equivalent financing terms—the AuD professional retains “just over half” (approximately 54%) of the discretionary income available to the SLP professional. In practice, this disparity is likely conservative. Elevated back-end DTI ratios increase borrowing risk, often resulting in higher interest rates, stricter underwriting requirements, or denial of credit, thereby further constraining real-world affordability for AuD graduates.

##### Debt Repayment Scenarios and Salary Requirements

To further contextualize the economic burden of graduate-level borrowing, repayment scenarios were modeled using a hypothetical graduate-only loan balance of $100,000 at a fixed interest rate of 7.05%. Fig. 3 and Table 4 present estimated repayment obligations under standard 10-, 20-, and 30-year amortization schedules. 12

**Table 4:** Estimated monthly payments, total repayment amounts, cumulative interest, and corresponding minimum annual salaries required to maintain an 8% student-loan debt-to-income (DTI) ratio for a $100,000 graduate-only loan at 7.05% interest under standard 10-, 20-, and 30-year repayment plans

		Student loans			Loan repayment estimates
Direct unsubsidized loan	Interest simple	Repay period (y)	Interest	Total amount	Monthly payment
$100,000.00	7.05	10	$39,639.60	$139,639.60	$1,163.66
$100,000.00	7.05	20	$86,792.73	$186,792.73	$778.30
$100,000.00	7.05	30	$140,299.58	$240,299.58	$668.66

Note: Salary estimates assume gross income and do not account for taxes, benefits, or other debt obligations.

**Fig. 3:**
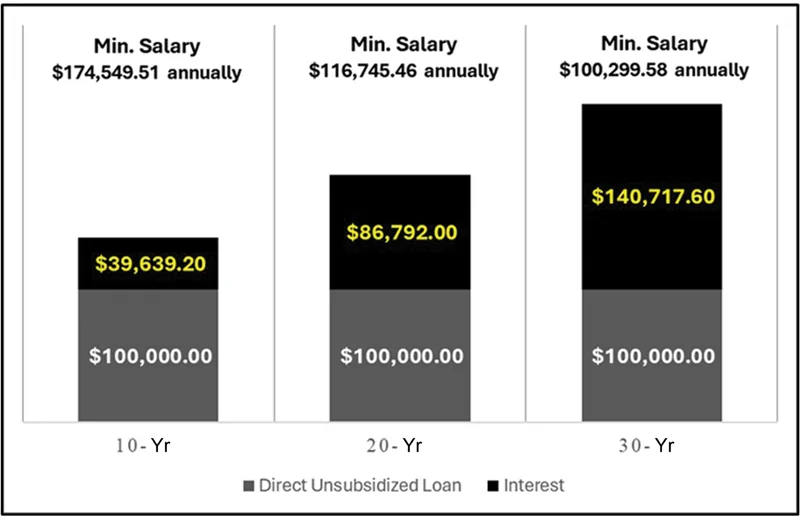
Minimum annual gross salary required to maintain a student-loan debt-to-income (DTI) ratio of 8% for a hypothetical $100,000 graduate-only loan at a fixed 7.05% interest rate under 10-, 20-, and 30-year standard repayment plans. Stacked bars represent the total repayment amount, partitioned into principal and cumulative interest. See text and Table 4 for repayment assumptions.

For interpretability and consistency with common affordability benchmarks, repayment capacity was evaluated using a target student-loan DTI ratio of 8% of gross income—a threshold frequently cited in financial planning literature as indicative of manageable educational debt. Under this assumption, the minimum annual salaries required to sustain repayment across the three repayment horizons are shown in Fig. 3 and summarized in Table 4 .

Across all repayment durations, the income required to maintain an 8% DTI substantially exceeds current earnings for many audiologists. The median annual wage for audiologists in 2023, as reported by the U.S. Bureau of Labor Statistics (BLS), was $87,740. 13 At this income level, none of the modeled repayment plans achieve the 8% affordability threshold. Instead, borrowers earning the median salary would face substantially higher student-loan DTI ratios, constraining disposable income and extending repayment timelines.

When repayment is calibrated to typical monthly payment levels affordable at the median audiologist salary, full amortization of a $100,000 loan would extend beyond “three decades”—approaching or exceeding the duration of many clinical careers. This finding underscores the structural divergence between educational debt loads and early- to mid-career earnings in audiology, particularly when compared with health professions that offer higher initial compensation, shorter training periods, or greater access to subsidized repayment pathways.

Collectively, these repayment scenarios highlight how standard borrowing levels—rather than extreme or atypical debt—can produce long repayment horizons and persistent financial strain for audiologists.

#### Impact of Educational Debt on Quality of Life, Workforce Sustainability, and Access to Care

High educational debt combined with modest early-career wages shapes the lived experience of early-career audiologists and produces downstream effects that extend beyond individual financial strain. These effects can be understood across four interrelated domains.

First, for many early-career audiologists, substantial student loan obligations coincide with rising housing costs, limited early wage growth, and delayed asset accumulation. This financial compression reduces disposable income during a critical developmental period and is associated with postponed life milestones, including home ownership, family formation, retirement saving, and practice ownership. Over time, sustained financial strain may influence job mobility, career planning, and perceptions of long-term professional viability.

Second, at the workforce level, educational debt influences who is able to enter, persist in, and remain economically stable within the profession. High educational costs and modest early-career earnings shape how prospective students evaluate the AuD pathway relative to other health professions with shorter training periods, higher initial salaries, or more predictable repayment trajectories.

National AuD enrollment patterns remain heterogeneous across institutions and regions, precluding simple conclusions about aggregate growth or decline. However, interview-based and survey studies suggest that educational cost, anticipated debt burden, and salary expectations are increasingly salient in how audiologists experience training and assess career sustainability. 14 15 16 17 18 Rather than functioning as a universal deterrent, financial pressures may act as a selective constraint, privileging individuals with greater financial support, prior wealth, or tolerance for prolonged economic tradeoffs. This dynamic has implications for socioeconomic diversity, geographic distribution, and long-term workforce resilience. Importantly, this selective attrition does not operate in a neutral environment: the workforce supply analyses presented earlier in this issue document a profession already failing to produce graduates at a rate sufficient to meet projected demand. Educational debt burden, operating as a filter on who can afford to enter and remain in audiology, functions as a structural accelerant of that documented supply shortfall—not merely a distributional concern, but a compounding constraint on aggregate workforce capacity.

Third, educational debt also interacts with geographic wage differentials to shape early-career practice location. BLS data demonstrate systematically higher wages in metropolitan labor markets compared with nonmetropolitan and rural regions. 13 For early-career clinicians managing substantial loan repayment obligations, higher-wage settings may be economically necessary to maintain financial stability. Yet even within higher-wage metropolitan markets, the growing penetration of third-party administrator (TPA) contracts and device-bundled reimbursement arrangements increasingly constrains per-encounter revenue—meaning that geographic relocation toward higher nominal wages does not fully insulate clinicians from the revenue compression that structural market forces are imposing across practice settings. The interaction between debt burden, geographic wage differentials, and TPA-mediated revenue ceilings compounds the financial pressure on early-career audiologists in ways that location choice alone cannot resolve.

In rural, low-income, and border regions—where wages are lower, private-pay opportunities are limited, and reliance on public insurance is greater—educational debt further constrains workforce supply. 19 As a result, longstanding patterns of geographic maldistribution persist, with shortages most evident in rural counties, low-income urban neighborhoods, Tribal and Indigenous communities, and federally designated Health Professional Shortage Areas. Debt burden, thus, indirectly narrows the range of practice settings that are financially viable for early-career audiologists. 18

Critically, the debt burden documented here does not exist in isolation from the professional classification issues examined elsewhere in this issue. As the taxonomy and scope-of-practice analyses in this volume demonstrate, audiology has not yet secured a reimbursable diagnostic identity that is independent of device dispensing—the structural floor that, in professions such as OPT, provides a revenue base decoupled from product sales. An optometrist or physician carrying educational debt comparable to that of an audiologist operates within a reimbursement environment that includes billable diagnostic services capable of sustaining doctoral-level wages; the audiologist, by contrast, practices within a model increasingly dominated by TPA pricing and device-bundled transactions that compress per-encounter revenue. This structural distinction explains why DTI misalignment in audiology is not simply a wage-level problem amenable to incremental salary growth, but a revenue-architecture problem that limits the ceiling of what audiology graduates can earn regardless of practice setting or clinical productivity. Reform of educational financing, in isolation, cannot resolve a problem that is also rooted in how the profession's clinical work is classified and compensated.

Fourth, economic strain often persists beyond workforce entry. Early-career audiologists frequently report debt-related stress alongside rising living costs and limited wage growth. Qualitative and mixed-methods studies associate this compression with reduced job satisfaction, heightened burnout risk, and increased consideration of job changes or alternative career pathways. 15 16 18 While precise attrition rates are difficult to quantify nationally, early-career instability may reduce the pool of clinicians who later assume roles as clinical preceptors, faculty, or professional leaders—placing additional strain on training capacity and mentorship pipelines.

These workforce dynamics have broader public health consequences. Untreated hearing loss is associated with increased risk of falls, social isolation, depression, reduced income, diminished quality of life, and cognitive decline. 20 When educational debt constrains workforce size, distribution, or specialty availability, access gaps widen—particularly for populations already at elevated risk. Financial pressures borne by individual clinicians thus aggregate into structural access limitations, reinforcing inequities and undermining audiology's contribution to population health.

#### Conclusion

Taken together, the economic burden associated with the AuD has emerged as a structural determinant of workforce sustainability—shaping not only the size of the profession, but also its geographic distribution, diversity, and clinical capacity. When elevated educational debt intersects with modest wage growth, constrained reimbursement, and rising population-level demand for audiologic services, the result is a workforce operating under increasing financial and professional strain.

ROI in a clinical doctorate is determined not only by the cost of education, but also by the revenue pathways available to graduates over the course of their careers. In audiology, educational costs and time-to-degree have increased substantially with the transition to the AuD, yet wages have not demonstrated a commensurate upward shift relative to peer health professions—or even relative to master's-level fields such as SLP. Discussion of longitudinal wage trajectories and earnings dispersion is examined separately in the article, Audiology at the Intersection of Labor, Revenue, and Professional Taxonomy, in this issue.

This persistent disconnect between training intensity, educational cost, and earning potential places structural strain on workforce stability, geographic distribution, and access to care. The findings presented here suggest that ensuring audiology remains accessible to future clinicians, financially sustainable for graduates, and responsive to patient and community needs will require coordinated action across educational institutions, professional organizations, and state and federal policy frameworks—addressing both the cost of training and the economic value of clinical practice.

As the preceding analysis makes clear, meaningful improvement requires progress on two parallel fronts: containing the cost of training and expanding the revenue architecture available to graduates. These are not independent problems—the latter is inseparable from the professional classification and reimbursement reform arguments developed elsewhere in this issue. The following section examines the financing mechanisms that underlie this economic burden and identifies opportunities for reform on both dimensions.

### Part II. Reducing Economic Burden: Understanding Types of Graduate Student Debt

Having established how educational debt shapes individual career decisions and the stability of the audiology workforce, this section examines the financing structures that generate and perpetuate that burden. Meaningful reduction of the economic pressure associated with audiology education requires a clear understanding of how graduate training is financed—and how those mechanisms differentially affect doctoral-level health professions.

This section reviews the primary federal, institutional, and private funding pathways available to graduate students, with emphasis on federal student aid policy. Particular attention is given to how historical classifications and recent policy proposals position audiology differently from other health professions—shaping not only the cost of education, but also the economic return of the AuD degree and, by extension, workforce sustainability.

#### Federal Student Aid

Federal student aid authorized under Title IV of the Higher Education Act of 1965 constitutes the primary source of financing for graduate education in the United States. Title IV has been amended multiple times—most recently in 2025 21 —to reflect evolving approaches to higher education finance, borrower protections, and federal lending structures. These provisions continue to govern the terms under which graduate and professional students access federal loans.

For students pursuing the AuD degree, two Title IV loan programs are most relevant: the Direct Unsubsidized Loan and the Direct Graduate PLUS Loan. Given the clinically intensive and full-time nature of AuD training, these programs function as the principal mechanism through which students finance tuition, fees, and living expenses during doctoral education.

##### Direct Unsubsidized Loans

Direct Unsubsidized Loans are not based on financial need and do not require credit underwriting. Graduate students are eligible to borrow up to $20,500 per award year. However, comparable health professions—including medicine, osteopathy, dentistry, OPT, veterinary medicine, podiatry, pharmacy, and selected graduate programs in public health and health administration—are eligible for higher unsubsidized loan limits.

These higher limits reflect historical participation in the now-discontinued Health Education Assistance Loan (HEAL) program. When HEAL was terminated in 1998, its lending capacity was effectively preserved by increasing unsubsidized loan limits for previously eligible programs. At that time, audiology remained a master's-level profession and was therefore excluded from HEAL eligibility. Despite the subsequent transition of audiology to a doctoral-level entry-to-practice model, eligibility criteria for higher unsubsidized loan limits have not been updated. As a result, AuD students remain restricted to lower annual borrowing limits despite a longer program duration and increased educational cost. This exclusion is not an isolated administrative oversight; it reflects the same pattern of professional misclassification that the taxonomy and scope-of-practice articles in this issue document across reimbursement policy and scope-of-practice regulation. The HEAL-era classification captured audiology at a moment before its transition to doctoral-level practice, and federal policy has never corrected for that transition—a failure with compounding financial consequences for every graduating cohort since. Financing disadvantage and classification disadvantage are, in this respect, the same structural problem expressed in different policy domains.

##### Direct Graduate PLUS Loans

Direct Graduate PLUS Loans—modeled after the Parent PLUS Loan program for undergraduate education—represent a second major source of federal borrowing for graduate students. Historically, Graduate PLUS borrowing has been permitted up to the full cost of attendance, making it a critical financing mechanism for students enrolled in high-cost programs with limited access to higher unsubsidized loan limits, including audiology.

As a result, AuD students frequently rely on Graduate PLUS Loans to bridge the gap between tuition, living expenses, and the federally capped Direct Unsubsidized Loan amount. However, Graduate PLUS Loans are more expensive over the life of repayment for two principal reasons. First, they carry higher origination fees, assessed as a percentage of the gross loan amount and deducted at disbursement, thereby reducing the net funds received while leaving the full principal subject to repayment. Second, Graduate PLUS Loans accrue interest at higher statutory rates than Direct Unsubsidized Loans, with interest calculated on the full principal from the time of disbursement. Together, these features significantly increase cumulative repayment costs for borrowers who depend heavily on Graduate PLUS financing.

Importantly, the financing landscape described above has operated differently for prior cohorts of audiology students than it is likely to operate for future entrants. As a result, prior cohorts of AuD students were able to finance high-cost training through relatively unrestricted access to Graduate PLUS Loans, allowing substantial educational debt to accumulate with limited upfront constraint. While this model transferred financial risk to graduates over time, it allowed enrollment decisions to proceed without binding constraints on borrowing.

The policy changes introduced under the One Big Beautiful Bill Act (OBBBA) represent a fundamental shift away from this approach. Rather than permitting high levels of debt with deferred consequences, the new framework imposes binding borrowing constraints at the point of entry, effectively forcing a reassessment of affordability, ROI, and creditworthiness before enrollment. This shift fundamentally alters the financing landscape for future audiology students, with direct implications for academic programs and the profession's workforce pipeline.

##### New Provision Under the One Big Beautiful Bill Act: Federal Loan Caps and Professional Degree Classification

At the time of this writing, the OBBBA authorizes a substantial structural change to federal student lending by permitting aggregate caps on total graduate borrowing across all federal loan programs, including Direct Unsubsidized and Direct Graduate PLUS Loans. 22 Under this framework, Graduate PLUS borrowing would no longer be available up to the full cost of attendance, but instead would be subject to program-level aggregate borrowing limits, with specific parameters to be determined through subsequent Department of Education rulemaking.

In addition to changes affecting Graduate PLUS borrowing, the OBBBA introduces new aggregate limits on Parent PLUS Loans—reportedly capped at approximately $60,000 per borrower. Prior to this legislation, Parent PLUS borrowing—like Graduate PLUS—was effectively uncapped up to the cost of attendance, subject only to credit approval. The introduction of a fixed Parent PLUS cap represents a substantive departure from prior federal lending policy and further constrains the ability of families to finance high-cost educational pathways.

For audiology, this change is particularly consequential. As federal borrowing options narrow for both students and parents, future AuD cohorts may face increased reliance on private loans—or may be deterred from enrollment altogether—particularly in the absence of concurrent reductions in program cost or improvements in postgraduation earning potential.

Current statutory language and accompanying guidance indicate that higher aggregate loan limits would be reserved for programs designated as professional degrees under federal statute. Audiology is not currently included among the programs identified in this classification, despite requiring doctoral-level education, state licensure, and extensive supervised clinical training. This exclusion, however, is currently unsettled. On June 24, 2026, the U.S. District Court for the District of Columbia preliminarily stayed the Department's narrowed professional-degree definition nationwide in American Association of Nurse Practitioners v. McMahon (consolidated with PA Education Association v. Department of Education*). 23 For the duration of the stay, the Department is treating an expanded interim list of programs–including audiology and speech–language pathology–as professional for loan-limit purposes, consistent with the Department's own finding in the Final Rule preamble that these programs satisfy the operative three–part regulatory test for professional–degree status. This interim treatment is provisional and subject to change as the litigation proceeds; it does not resolve the underlying regulatory definition, which remains the subject of active dispute.

Key policy elements relevant to audiology include:

- Establishment of aggregate federal borrowing caps for graduate and professional students.
- Application of these caps to graduate PLUS loans.
- Preservation of higher aggregate caps for historically recognized professional degree programs.
- Proposed exclusion of audiology from the federal definition of professional degree programs, absent further regulatory or legislative clarification, currently stayed by federal court order pending litigation (see above).

Aggregate borrowing caps are not inherently flawed as a policy instrument. In principle, such limits may improve long-term affordability by preventing debt accumulation that is misaligned with expected earnings. In professions where educational cost, time-to-degree, and wage trajectories are reasonably aligned, borrowing caps may function as a safeguard against unsustainable DTI ratios.

However, in audiology—where tuition levels, program length, and clinical training requirements remain high relative to modest early-career wages—borrowing limits imposed without concurrent cost containment or professional reclassification may shift the financial burden rather than reduce it. In the absence of structural reform, such limits may constrain access to the profession or increase reliance on private lending, with implications for workforce sustainability and equity.

Beyond borrowing caps, emerging federal accountability frameworks, including debt-to-earnings (D/E) calculations under 34 CFR §668.403, further formalize the linkage between educational cost and graduate earnings. Programs with high median debt relative to earnings may face heightened regulatory scrutiny and potential eligibility consequences under evolving federal accountability standards. These developments underscore the urgency of aligning AuD program costs with sustainable wage trajectories.

##### Interest Accrual and Policy Changes

Federal student loan interest rates reset annually for loans first disbursed on or after July 1 and are determined by the high yield of the 10-year Treasury note at the final auction prior to June 1, plus a fixed statutory add-on. Graduate PLUS Loans consistently carry a higher add-on than Direct Unsubsidized Loans, resulting in higher interest rates and greater total repayment costs.

These costs were further amplified by the elimination of need-based Subsidized Loans for graduate and professional students, effective July 1, 2012. Prior to this change, subsidized loans did not accrue interest during enrollment or the postgraduation grace period. With their removal, interest now accrues on all federal graduate loans from the time of disbursement, substantially increasing total repayment obligations by the time graduates enter the workforce (with the exception of the temporary 0% interest and administrative forbearance period from March 2020 to August 2023).

In the context of the OBBBA, these interest dynamics take on heightened significance. Aggregate borrowing caps and constraints on Graduate PLUS availability may require students in excluded programs—such as audiology—to rely on higher-interest borrowing earlier in training or to seek private loans with fewer protections.

##### Title VII Health Professions Loans and Scholarships

In addition to Title IV, federal programs authorized under Title VII of the Public Health Service Act 24 provide financial support to students in select health disciplines through mechanisms such as Health Professions Student Loans, Loans for Disadvantaged Students, and Scholarships for Disadvantaged Students. These programs offer favorable features, including fixed 5% interest rates, interest subsidies during enrollment, and need-based grant funding.

Title VII programs are available to students in dentistry, pharmacy, podiatry, OPT, and veterinary medicine. Despite audiology's transition to a doctoral-level health profession, AuD students remain ineligible. This exclusion represents a persistent policy misalignment that results in higher borrowing costs for audiology students relative to peers in comparable health professions.

##### Private Student Loans and Alternative Financing

Students who exhaust federal aid options may turn to private education loans or income-share agreements. While these mechanisms can supplement funding, they typically carry higher long-term risk due to limited borrower protections, exclusion from income-driven repayment and forgiveness programs, and exposure to variable interest rates.

Other funding sources—such as graduate assistantships, federal work-study, and military health scholarships—can substantially reduce or eliminate educational debt but are constrained by program intensity, institutional priorities, competitiveness, or service obligations, limiting their scalability as system-wide solutions.

#### Conclusion

Part II demonstrates that the current financing landscape places audiology students at a structural disadvantage relative to peers in other health professions. Reliance on federal borrowing—particularly Graduate PLUS Loans—combined with lower unsubsidized loan limits, higher interest accrual, exclusion from Title VII programs, and limited access to subsidized graduate aid results in elevated lifetime repayment costs and weaker economic returns for AuD graduates.

Historically, relatively unrestricted access to Graduate PLUS borrowing allowed high-cost educational models to persist, with financial risk deferred to graduates over time. The policy changes introduced under the OBBBA, including aggregate borrowing caps and new constraints on Parent PLUS Loans, represent a fundamental shift. Rather than permitting high debt with delayed consequences, the new framework places binding limits at the point of entry, intensifying scrutiny of affordability, D/E alignment, and professional classification.

Emerging federal accountability mechanisms, including D/E standards, further formalize the linkage between educational cost and graduate income. In the absence of alignment between program cost, financing structures, and wage trajectories, these developments may constrain access to the profession, increase reliance on private lending, and heighten regulatory exposure.

Recognizing these financing inequities is essential for informing institutional reform, guiding policy advocacy, and developing strategies that reduce economic burden while strengthening income pathways. Crucially, neither cost containment nor revenue expansion is sufficient alone: an educational model that becomes cheaper without becoming more remunerative will not resolve the ROI problem that constrains workforce entry and retention. Part III examines how institutional, federal, and state levers can be aligned to address both dimensions simultaneously.

### Part III. Strategies to Reduce Economic Burden and Strengthen Workforce Sustainability

Having established the economic pressures embedded within audiology education—and their downstream effects on workforce supply, geographic distribution, and patient access—it is necessary to move from diagnosis to intervention. Reducing the financial burden borne by AuD students and early-career clinicians is central not only to improving individual ROI, but also to strengthening the long-term sustainability and professional autonomy of the field.

Effective reform must operate across multiple, interconnected levels. Institutional and programmatic decisions shape the immediate cost of training; federal and state policy determine the structure and affordability of financing; and the fourth-year externship represents a critical inflection point where educational design, workforce deployment, and economic efficiency intersect. Accordingly, this section outlines three complementary domains for action: (1) program- and institution-level strategies to reduce educational cost and improve efficiency; (2) federal and state policy levers to modernize financing and align incentives with workforce needs; and (3) reform of the fourth-year externship as a high-impact opportunity to reduce debt accumulation while strengthening clinical readiness.

#### Program- and Institutional-Level Strategies

Institutions play a direct role in shaping the total cost of audiology education. While many drivers of tuition are structural, targeted programmatic reforms can meaningfully reduce student borrowing, improve equity, and enhance the financial feasibility of the AuD pathway.

#### Tuition Models and Program Cost Transparency

Graduate tuition structures vary widely across institutions and often lack alignment with program costs or market-based earnings. More transparent pricing models—including differential tuition, cost-of-delivery analyses, and predictable multi-year tuition schedules—can help students make informed decisions and reduce surprise increases during their training.

Programs may also explore structural alternatives to conventional tuition models, including flat-rate tuition for clinical years that reflects reduced didactic load, bundled fee structures that eliminate ancillary charges, and public–private partnerships that allow reduced tuition in exchange for defined service commitments. Accelerated 3-year tracks are another emerging model, though these vary considerably in design: some compress delivery for high-performing or nontraditional students without reducing curricular content, while others achieve the shorter timeline by reducing instructional hours or clinical training requirements as a cost-containment measure. The latter approach risks trading tuition savings for weaker preparation, and any evaluation of 3-year models should distinguish between genuine efficiency gains and simple curriculum reduction.

##### Scholarships, Stipends, and Assistantships

Most AuD programs provide limited systematic financial support beyond federal loans. Institutions can reduce reliance on borrowing through graduate assistantships designed around AuD clinical schedules, donor-supported scholarships targeting first-generation and underrepresented students, institutional grants linked to community or clinical outreach, and employer-sponsored tuition pipelines with postgraduation employment commitments.

##### Clinical Education Innovations

Institutions can reduce student cost and improve efficiency by integrating the first year of the AuD curriculum into the final year of undergraduate study, developing regional clinical training consortia, adopting hybrid or tele-supervision models to reduce relocation and travel expenses, and expanding high-fidelity simulation in the preclinical years to reduce reliance on unpaid external placements.

A potential complementary strategy involves enabling provisional or trainee-level Hearing Instrument Specialist licensure, where permitted by state law. When structured appropriately, this pathway allows supervised, compensated clinical work that offsets educational expenses, expands clinic capacity, and enhances applied skill development—without supplanting AuD-level training requirements.

##### Reforming the Fourth-Year Externship: A High-Impact Opportunity

The fourth-year externship represents the single largest structural contributor to the cost of the AuD degree—and one of the most actionable opportunities for reform. In many programs, students pay full tuition while working full time in revenue-generating clinical roles, receiving the majority of their supervision from externally licensed preceptors who typically receive no direct compensation from the university. This structure imposes a triple financial burden on students: ongoing tuition obligations, continued living expenses, and foregone earnings during a year of full-time clinical labor.

From both an educational and ethical standpoint, this arrangement reflects an incongruence between educational cost, instructional delivery, and financial accountability. While universities retain responsibility for academic oversight, much of the instructional labor during the externship year is delivered outside the institution, raising questions of transparency, value, and equity in clinical doctoral education.

Transitioning toward paid externship models—which are standard across several comparable health professions—offers a clear pathway to reducing student debt while strengthening workforce outcomes. Compensated externships can reduce reliance on high-interest federal and private borrowing, improve equity for students without external financial support, enhance recruitment into underserved and high-need clinical settings, and establish clearer professional expectations during advanced clinical training.

Importantly, paid externship models are not without precedent in audiology. Prior to the transition to the AuD as the entry-to-practice degree, audiology training followed a model in which academic preparation was completed at the graduate level and intensive clinical training occurred postdegree through structured fellowships or supervised practice—analogous to the clinical fellowship year in SLP or residency models in medicine. Under such frameworks, trainees were recognized as early-career professionals rather than students and were typically compensated for their clinical labor.

Revisiting elements of this approach—without abandoning doctoral-level preparation—offers a viable pathway for reform. A postdegree, paid clinical fellowship or residency-style model would allow universities to concentrate tuition-supported education on didactic and preclinical training, while shifting advanced clinical training into compensated, workforce-integrated roles. Such a model would more closely align educational cost with institutional instruction, reduce tuition reliance during the most clinically productive year of training, and accelerate entry into salaried practice.

Paid externship or residency-style models also address sustainability on the preceptor side. The current reliance on uncompensated instructional labor contributes to preceptor fatigue, limits site availability, and constrains clinical training capacity. Formal compensation, academic appointments, or health-system partnerships offer mechanisms to stabilize and expand high-quality training sites while recognizing the value of clinical supervision.

In parallel, programs may pursue externship-year tuition reform to better align student cost with institutional service delivery. Potential approaches include supervision-only tuition models, reduced or fixed-rate ‘clinical year’ tuition, or university-funded stipends offset through clinic affiliation agreements. Tuition waivers are a further option, though their design depends on institutional priorities: some programs may apply waivers as relief for students in unpaid placements to offset the absence of earned income, while others may use waivers as an incentive to expand the supply of paid externship sites by rewarding students who secure compensated placements. Accrediting bodies and licensure boards can further support these efforts by incentivizing compensated placements, promoting university–health system partnerships, and aligning advanced clinical training expectations with documented workforce needs.

In conclusion, externship and postdegree clinical training reform—whether through paid externships, residency-style fellowships, or hybrid models—has the potential to reduce graduate borrowing by tens of thousands of dollars while improving clinical training quality, expanding workforce capacity, and reinforcing the economic, ethical, and professional foundations of a doctoral-level audiology workforce.

##### Federal Policy Levers for Loan Reform

While institutional reforms can reduce immediate costs, durable improvement in affordability requires policy alignment at the federal and state levels.

- Modernizing federal loan structures

As outlined in Part II, federal loan policy has not kept pace with audiology's transition to a doctoral-level entry-to-practice degree. Proposed changes under the OBBBA risk formalizing this disconnect by excluding audiology from professional-degree classifications that receive higher aggregate borrowing limits, though this exclusion is presently stayed pending litigation and could still be reinstated depending on the outcome.

Policy pathways that would improve alignment include revising the federal definition of professional degree programs to include audiology, adjusting aggregate borrowing caps to reflect program length and required clinical training, reinstating interest-subsidized borrowing for extended clinical doctorates, and reducing the interest rate differentials and origination fees that disproportionately affect excluded professions.

Absent such alignment, borrowing constraints may limit access to the profession or accelerate reliance on private lending—without addressing underlying cost and wage dynamics.

- Policy-induced cost alignment pressures

As described in Part II, the OBBBA's aggregate borrowing caps function as de facto price ceilings on programs that historically relied on uncapped Graduate PLUS and Parent PLUS loans, with total federal borrowing for many AuD students potentially capped near $100,000. Emerging D/E accountability frameworks further formalize this pressure: programs with high median debt relative to earnings may face regulatory consequences, intensifying scrutiny of economic viability. Evidence from prior workforce analyses suggests that current debt levels are already misaligned with earning capacity, with some AuD graduates exhibiting DTI ratios exceeding 1.5—levels widely regarded as financially unsustainable. Under these conditions, continued reliance on increased borrowing is unlikely to improve affordability or workforce stability.

Consequently, reform must address two reinforcing constraints: the cost of training, which must be brought into alignment with new federal borrowing limits; and the revenue ceiling that current professional classification imposes on graduates regardless of practice setting or productivity. Federal loan caps, in the absence of this dual alignment, risk transferring the financial burden to private lending markets, parents, or prospective students—rather than correcting the structural imbalance between educational cost and professional earnings.

- Interest rate and origination fee reform

Even when borrowing access exists, the cost of borrowing remains a central determinant of affordability. Graduate PLUS interest rates, origination fees, and the elimination of in-school subsidies function as structural amplifiers of debt burden.

Targeted reforms could include harmonizing interest rates across doctoral-level health professions, reducing or eliminating origination fees, reinstating interest subsidies during extended clinical training, and providing dedicated interest relief during the fourth-year externship.

These measures would reduce lifetime repayment costs without expanding total borrowing and would strengthen the economic viability of the AuD pathway under emerging federal accountability standards.

- Forgiveness and service incentives

Audiology is well positioned for inclusion in federal workforce programs designed to address geographic and specialty-based access gaps. Loan forgiveness and service-linked incentives can reduce early-career financial pressure while directing clinicians toward high-need settings.

Potential policy pathways include expanding Public Service Loan Forgiveness eligibility to encompass a broader range of clinical environments, including private practices serving Medicaid populations; establishing audiology-specific service corps loan repayment programs targeting rural, tribal, and medically underserved areas; extending National Health Service Corps eligibility to include audiology; and creating federal tax incentives for employers that provide structured loan repayment benefits.

Properly designed, such initiatives would simultaneously improve affordability for graduates and enhance access to care in underserved communities.

##### State-Level Incentives

State governments can play a complementary role by aligning educational financing with documented workforce shortages. Targeted state-level incentives may include loan repayment or forgiveness programs for audiologists practicing in designated shortage areas, tuition reimbursement or grant programs tied to in-state service commitments, state-funded paid externships or residency-style placements within public hospitals, Veterans Affairs facilities, schools, and rural health systems, and matching-fund scholarship programs that leverage state appropriations alongside institutional, philanthropic, or employer contributions.

Unlike broad federal reforms, state-level initiatives can be tailored to local workforce distribution challenges and population needs. When strategically deployed, these mechanisms can reduce graduate debt burden while strengthening in-state workforce retention and specialty capacity.

#### Conclusion

Reducing the economic burden associated with the AuD degree requires coordinated, multi-level action. Institutional reforms can improve cost transparency and align tuition with instructional value; federal and state policy changes are necessary to modernize financing structures and correct misclassification; and reform of the fourth-year externship represents one of the most immediate opportunities to reduce borrowing while strengthening clinical readiness.

At the same time, durable improvement in ROI requires alignment between educational cost and professional earnings. Without parallel progress in both cost containment and revenue generation, borrowing constraints alone will not resolve affordability challenges.

Together, these strategies form a pragmatic roadmap for strengthening ROI, stabilizing the workforce pipeline, and supporting geographic and specialty distribution aligned with public health needs. Absent such reform, financial pressures will continue to constrain workforce growth, access to care, and professional autonomy. With intentional alignment, audiology can advance toward a training and financing model that is economically sustainable, workforce-stable, and consistent with the responsibilities of a modern doctoral-level health profession.

### Part IV. Conclusion and Future Directions

The AuD's establishment as the entry-to-practice degree strengthened clinical preparation and advanced audiology's alignment with other doctoral-level health professions. Yet the prevailing educational and financing model has produced an economic burden that increasingly threatens the attractiveness, diversity, and long-term sustainability of the audiology workforce. Rising tuition, modest early-career wages, and misalignment within federal student aid policy weaken the ROI of the AuD degree and, in turn, constrain workforce supply, geographic distribution, and access to hearing and balance services.

This article has identified three interrelated domains shaping the economic viability of audiology education: (1) the broader economic context and workforce effects of student debt; (2) financing structures that position audiology at a relative disadvantage compared with peer health professions; and (3) actionable institutional and policy strategies to reduce economic burden and improve alignment. Progress will require coordinated engagement among academic programs, professional organizations, policymakers, employers, and accrediting bodies—not only to implement reforms, but also to monitor outcomes and recalibrate as federal policy, healthcare delivery models, and workforce demands evolve.

The objective is not merely to reduce the cost of audiology education, but to ensure that the financial investment required to become an audiologist is proportionate to clinical responsibility, training intensity, and societal value. The evidence presented here is consistent with divergence across educational cost, financing mechanisms, and professional earnings suppressing the economic conditions necessary for professional autonomy—and with alignment across these elements being a necessary, though not sufficient, condition for its realization.

Ultimately, professional autonomy in audiology cannot be realized—or sustained—without an educational and financing framework that is economically viable for those entering the profession. Aligning training cost, revenue generation, and federal policy is, therefore, not ancillary to workforce strategy; it is foundational to audiology's future as a self-determined healthcare profession. This financing disadvantage is not independent of the taxonomic constraints documented elsewhere in this issue; it is a downstream expression of the same unresolved classification gap, in which audiology's post-2007 doctoral transition was never reflected in the federal definitions–CMS reimbursement categories, BLS occupational classification, or Title IV/VII professional–degree designations–that determine both earning capacity and borrowing terms.

Several research and policy priorities emerge from this analysis. First, the profession needs prospective tracking of AuD enrollment and attrition patterns under emerging OBBBA borrowing constraints—to determine empirically whether aggregate caps are deterring qualified applicants or accelerating the supply shortfall documented in earlier articles of this issue.

Second, longitudinal studies linking individual debt burden to geographic practice choice, specialty selection, and career duration remain largely absent from the audiology literature; such evidence would move the field beyond inference and toward targeted intervention. Third, the externship compensation question warrants formal economic modeling: what would paid residency–style models cost programs and health systems, and what would they return in workforce capacity, preceptor stability, and reduced graduate debt? The health economics framework developed in the companion article in this issue offers a productive starting point for that modeling. Finally, federal classification reform—specifically, the inclusion of audiology within professional–degree designations for loan limit and Title VII purposes—requires coordinated advocacy grounded in the evidentiary record this issue has assembled. The workforce roadmap developed in the concluding article of this volume identifies the governance structures and professional leadership mechanisms through which that advocacy can be organized and sustained.
